# Vegetative propagation of mosses under controlled conditions: A reproducible, low-cost method for sustainable moss cultivation^☆^^☆☆^

**DOI:** 10.1016/j.mex.2026.103858

**Published:** 2026-03-11

**Authors:** Liga Strazdina, Lilita Abele, Roberts Krumins, Juta Karklina, Gederts Ievinsh, Inga Straupe, Edgars Karklins

**Affiliations:** a University of Latvia, Botanical Garden, Riga, Latvia; b Latvia University of Life Sciences and Technologies, Jelgava, Latvia; c Bulduri Technical School, Biotechnology Center, Jurmala, Latvia; d University of Latvia, Faculty of Medicine and Life Sciences, Riga, Latvia

**Keywords:** Bryophyte cultivation, Moss propagation, Green infrastructure

## Abstract

This protocol presents a reproducible, sustainable method for vegetative propagation of mosses under controlled but non-sterile conditions. It enables scalable cultivation of mosses for green infrastructure while reducing dependence on wild collections. The method is based on a fundamental and unavoidable trade-off: maintaining high humidity, which is essential for moss growth, while simultaneously limiting fungal and microbial contamination. This balance determines success, restricts species selection, and defines facility and management requirements. All stages of cultivation and validation must therefore consider this constant challenge: promoting moss vitality while preventing overgrowth of competing microorganisms.

Compared to traditional sterile cultivation, this open-system method lowers costs and technical complexity without compromising growth efficiency. Four moss species – *Dicranum scoparium, Plagiomnium affine, Pleurozium schreberi,* and *Hypnum cupressiforme* – were successfully propagated on hemp mats under controlled light, humidity, and temperature regime. After four months, moss coverage exceeded 80% on most 60 × 60 × 0.4 cm tiles, demonstrating the method’s reproducibility and efficiency.

1. The protocol provides a cost-effective, replicable method for moss cultivation under controlled conditions in non-sterile, open environments.

2. The method minimizes ecological impact by reducing wild moss harvesting.

3 The method supports sustainable applications of mosses in green infrastructure and environmental biotechnology.

## Graphical abstract

**Figure:**
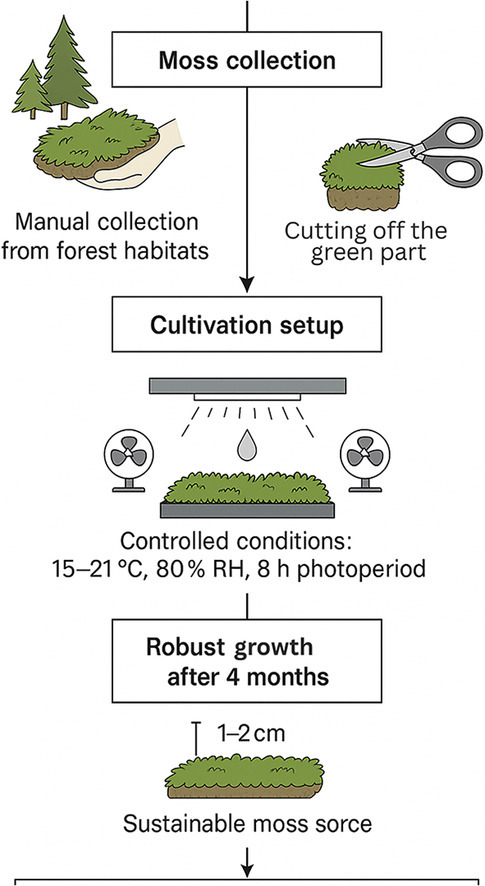

## Specifications table

**Table:** 

**Subject area**	Agricultural and Biological Sciences
**More specific subject area**	Indoor moss cultivation and propagation under controlled conditions for urban greening applications
**Name of your protocol**	Reproducible Indoor Cultivation of Mosses under Controlled Conditions
**Reagents/tools**	1. Moss starter material (preferably feather mosses, e.g., *Hypnum cupressiforme, Pleurozium schreberi, Brachythecium rutabulum, Cirriphyllum piliferum, Hylocomiadelphus triquetrus*); 2. Substrate material: hemp fiber mats; 3. Fogging irrigation system; 4. Environmental control equipment: temperature and humidity sensors, thermostats, LED grow lights with adjustable spectrum; 5. Hygrometer and thermometer; 6. pH meter for substrate monitoring; 7. Lux meter for measuring light (Soonkoda S8608 Digital Illuminance Light Meter or a similar model); 8. For irrigation, Type III reverse osmosis water (exhibiting electrical conductivity of 35 µS·cm⁻¹ and total dissolved solids (TDS) of 15 mg·L⁻¹ at 25°C) filtrated through semipermeable membrane was used.
**Experimental design**	Moss fragments are placed on moist hemp mats in climate-controlled conditions (15–21°C, 60–80% RH, 1102–2573±440 lux, PPFD 14.72–33.77±5.16 μmol s⁻^1^ m⁻^2^). Moss growth, vitality, and surface coverage percentage were estimated solely through direct visual assessment, without the use of digital image analysis or automated sensors.
**Trial registration**	Not applicable
**Ethics**	Not applicable
**Value of the Protocol**	• Offers an accessible, scalable alternative to sterile moss propagation. • Supports sustainable sourcing of mosses for use in green roofs, living walls, and other green infrastructure applications. • Establishes key climatic and operational parameters for optimizing moss growth in controlled indoor environments.

## Background

Mosses are increasingly utilized in green infrastructure (GI) design to enhance urban environmental quality and livability [1]. Their small size, poikilohydric physiology, and ability to absorb pollutants directly from the atmosphere make them efficient bioindicators and potential biofilters in urban ecosystems [2]. Since the pioneering work of Rühling and Tyler (1968) [3], which demonstrated mosses’ capacity to accumulate airborne heavy metals, they have been widely applied in biomonitoring and, more recently, in development of moss-based air filtration systems [4,5]. In several European and Asian cities, engineered moss modules such as “CityTree” and “MosSkin” have shown effectiveness in reducing fine dust and regulating urban temperatures [6]. These examples highlight the role of mosses in nature-based solutions to improve urban resilience and public health.

To meet the increasing demand for mosses, cultivation under controlled conditions is essential, as harvesting from natural habitats is neither sustainable nor ecologically responsible. Although mosses are frequently propagated *in vitro* under sterile laboratory conditions (e.g. [7]), numerous studies also report successful propagation in outdoor or greenhouse environments, particularly for *Sphagnum* species used in peatland restoration (e.g. USDA Forest Service protocols [8]). These outdoor approaches demonstrate the effectiveness of non-sterile, low-cost propagation systems and provide useful methodological parallels. However, they are less suitable for applications requiring precise control over moisture, temperature, and light – parameters that strongly influence biomass quality and survival during later transplantation to urban green roofs and walls. For this reason, the present protocol focuses on indoor propagation in open (non-sterile) controlled-environment facilities, which offers enhanced standardization and year-round production while maintaining low infrastructure requirements. This approach is significantly more accessible and cost-effective. This method is particularly suitable for stakeholders involved in large-scale applications, such as the development of green walls, green roofs, and other urban greening systems.

An additional rationale for indoor propagation is the need for acclimation. Mosses intended for urban GI installations must transition from controlled growth conditions to harsh rooftop or vertical-surface environments characterized by rapid wet-dry cycles, high irradiance, temperature extremes, and substrate instability. Prior acclimation improves stress tolerance and survival during deployment, as suggested by studies on bryophyte desiccation physiology and light adaptation [9,10]. Therefore, understanding the climatic parameters that support moss growth and pre-conditioning is essential for successful establishment in applied GI systems.

This protocol outlines the key environmental requirements and methodological considerations for indoor moss propagation under controlled, open-facility (non-sterile) conditions. Building on previous research [11], which demonstrated the potential of bryophytes as practical components of linear barriers for PM₂.₅ mitigation in urban landscapes, the protocol provides a sustainable, replicable method for producing high-quality moss biomass.

The protocol’s deliberate abandonment of axenic (sterile) growing conditions is its main innovation. Traditionally, microbial contamination is considered a failure, but in this context, it becomes a manageable variable. This approach is pragmatic and aligned with GI's goals, as the final product – the moss carpet – will be used in a non-sterile urban environment. Thus, the challenge shifts from preventing contamination to managing a microecosystem in which mosses have a competitive advantage over unwanted microorganisms.

Recent advances in moss propagation have emphasized vegetative reproduction through fragmentation and regrowth of protonemal or gametophytic tissues. For example, *Sphagnum* species have been propagated both axenically with rapid biomass accumulation [12] and under greenhouse or outdoor conditions with standardized fertilization and moisture regimes [8], while desert species such as *Syntrichia caninervis* have shown efficient regeneration on peat-pellet substrates [13]. These studies collectively confirm that multiple environmental parameters – light intensity, humidity, temperature, and substrate composition – play critical roles in determining moss viability and growth rate [14]. Their findings support the relevance of the environmental factors listed in this protocol, as all have been empirically shown to influence either biomass accumulation, regeneration speed, or structural quality in bryophyte cultivation. Accordingly, the protocol serves as a bridge between laboratory-based research and practical urban applications, enabling reproducible biomass production that supports both experimental consistency and real-world implementation of GI.

## Description of protocol

The protocol is presented as accessible because it does not require expensive sterile equipment, although its technical requirements are non-trivial. Successful implementation requires a "climate-controlled room", "LED grow lights", "fogging irrigation system" and, most importantly, "reverse osmosis water" and "modified Knopp medium" for fertilisation. It is also known that rainwater can be used successfully for watering mosses; however, in the present study, the amount of rainwater was insufficient and therefore was not analysed in detail.

The requirement for reverse osmosis (RO, a standard multi-stage reverse osmosis water filtration system typically used for hydroponics/aquariums) water is a critical detail that significantly affects the protocol's real-world cost and repeatability. Tap water contains variable amounts of minerals (e.g., calcium and magnesium) and disinfectants (e.g., chlorine). These substances can alter the pH of the substrate and directly inhibit moss growth, as mosses have adapted to low-nutrient and often acidic environments. Thus, the use of RO water is not just a recommendation; it is a must to ensure consistent results and avoid chemical stress on mosses. This means that anyone wishing to implement this protocol must invest in an RO filtration system, which adds capital investment and operational complexity that is not immediately apparent.

This protocol involves the vegetative propagation of four moss species – *Dicranum scoparium, Plagiomnium affine, Pleurozium schreberi*, and *Hypnum cupressiforme*. These species were selected based on the authors’ previous experimental results [11], which demonstrated their consistent performance under controlled cultivation, as well as practical consideration related to their ecology and morphology. All four species are widespread, ecologically cosmopolitan, and common across a broad range of natural habitats, making them easy to locate and collect and ensuring that the protocol can be reproduced independently of local site-specific flora. Moreover, they possess structurally robust gametophytes and show high physiological tolerance to fluctuating moisture, light and temperature regimes, properties that facilitate both fragmentation-based propagation and reliable establishment on artificial substrates. In addition, the chosen species represent different branching architecture – covering both acrocarpous and pleurocarpous growth forms – thus enabling the evaluation of the protocol across divergent morphological strategies relevant to substrate adhesion and shoot proliferation. This combination of ecological ubiquity, physiological resilience and morphological diversity was intentionally targeted. It is hypothesized that these characteristics are essential for establishing a mechanically stable moss layer capable of withstanding irregular hydration, thermal stress and other dynamic environmental conditions typical of urban settings. This procedure consists of three main stages: (1) collection of mosses from natural habitats, (2) processing of mosses and preparation of growth substrates, and (3) cultivation under controlled climatic conditions.

***Step 1: Collection of mosses from natural habitats***

**Materials:** gloves, 15 L plastic trays used as transport containers, and a collection permit from local authorities or the forest owner.

Moss samples were collected manually from forest habitats. Excess material such as pine needles, branches, and cones, was removed by gentle hand brushing. Moss clumps were carefully detached from the forest floor and placed into containers for transport. To minimize ecological disturbance, gaps left in the forest bed were filled with adjacent mosses. To reduce localized impact and maintain species diversity, samples were collected from multiple sites.

***Step 2: Processing mosses and preparation of growth substrates***

**Materials:** gloves, scissors, hemp mats, reverse osmosis water.

**Fig. 1:**
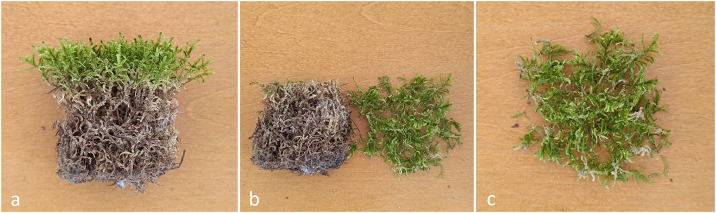
Preparation of *Pleurozium schreberi* samples for cultivation. Collected moss material (a) was divided into two portions (b), separating the green, actively growing shoots from the decayed basal parts; the green fraction (c) was used for cultivation.

Collected mosses were divided into two parts: the vital green (photosynthetically active) portion and the lower brown, decayed portion. The decayed material was trimmed away using scissors, leaving only the viable portion for further use in cultivation (Fig. 1). This separation was performed for all species to reduce the amount of decomposing organic matter in subsequent growth stages. Residual organic debris, such as pine needles, bark fragments, or branches, was removed to ensure clean propagation material.

**Fig. 2:**
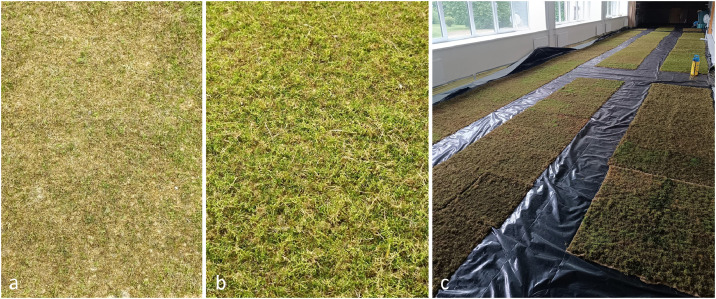
Comparison of *Pleurozium schreberi* cultivation density on hemp mats: (a) insufficient density; (b) sufficient density; (c) growing room with hemp mats placed on a plastic-covered floor to protect the material from debris.

Mosses were processed immediately after harvesting; if immediate processing was not possible, the material was stored in 15 L plastic trays at room temperature for no longer than five days to avoid desiccation and deterioration. Before application onto the substrate, the mosses were moistened with reverse osmosis water by gently squeezing them several times – similar to sponge – until the fragments were fully saturated. Because the harvested moss was heterogeneous and the mass of the applied material was not standartised gravimetrically, mosses were distributed manually over pre-wetted non-woven hemp fiber mats (60 × 60 × 0.4 cm) until the growing material was barely visible through the layer. Even distribution was achieved by hand, and the effective planting density was determined visually based on the proportion of substrate coverage. Preliminary observations indicated that approximately 50–90% initial coverage was insufficient, as mosses at these densities tended to dry, yellow and lose vigor. By contrast, near-complete (90–100%) coverage resulted in visibly greener and more vigorous growth, indicating that sufficient initial density was critical for preventing edge desiccation and promoting uniform establishment (Fig. 2).

Hemp fiber is a sustainable, biodegradable material with good water-retention properties, which are essential for maintaining the moisture regime of mosses [15]. However, it should be noted that hemp fiber itself is an organic material and is subject to decomposition processes and mould growth, especially in conditions of constant moisture. This means the substrate can become an additional source of contamination, potentially affecting the long-term success of cultivation.

Because the moss mass was not quantified, the exact biomass input per tile cannot be reported. Likewise, it is difficult to specify the number of replicates per species, as tiles were prepared with varying compositions: some contained a single moss species, others included two species in equal proportions, and some consisted of mixed, non-standardized assemblages combining several species in random ratios, covering 200 hemp tiles in total (each tile measured 0.36 m^2^). This variability reflects the exploratory nature of the protocol development phase. The prepared hemp mats with mosses were then transferred to a climate-controlled facility for further cultivation. Hemp mats were placed directly on a plastic-covered floor (Fig. 2) to prevent contact with the underlying surface and to reduce unintended contamination from soil particles or debris.

***Step 3: Cultivation under controlled climatic conditions***

**Materials:**

• Growing room (124 m^2^), where the floor was covered with waterproof plastic and hemp tiles with mosses were placed horizontally on the floor (Fig. 2)
• Reflective thermal insulation foil (3 mm thick, laminated on one side)
• Waterproof plastic film for floor covering
• LED projectors (200 W, 17,600 Lm, bright white 6500 K)
• Household fans
• 15 L electric water pulverizer

### Climate parameters in the growing room

**Temperature.** Maintained between 15–21°C, as higher temperatures promote mould development [14]. Limiting the temperature to 21°C has been identified as an essential measure to suppress mould growth, as higher temperatures and humidity promote its rapid spread.

**Lighting.** A combination of sunlight from the northern windows supplemented with white LED panels and fluorescent lights provided 1102–2573±440 lux, PPFD 14.72–33.77±5.16 μmol s^⁻1^ m^⁻2^ at moss level, with an 8 h light / 16 h dark photoperiod (9:00–17:00).

**Humidity.** Relative humidity was maintained at approximately 80%. During irrigation, humidity temporarily increased to ensure adequate moisture. Air humidity cycle was introduced: 80% during the day and 60% at night. This is not a random regime, but a deliberate attempt to mimic natural conditions (such as dew formation and subsequent drying). This approach is a way to control pests without using chemical means passively. Mosses, being poikilohydric organisms, can withstand periods of drought, while many moulds require constant high humidity to thrive.

**Ventilation.** Air circulation was maintained using household fans. Proper ventilation prevented mould growth, while excessive airflow was avoided to prevent moss desiccation. Outdoor air exchange ensured an adequate supply of carbon dioxide (CO_2_) for photosynthesis. Fans were turned off during irrigation (9:00–16:00) and turned on overnight (16:00–9:00) on medium-high settings so that mosses would be relatively dry in the morning.

**Irrigation and fertilization.** Mosses were misted three times per workday (9:00, 12:00, and 15:00; Monday–Friday) with reverse osmosis water using an electric pulverizer. Each misting cycle used 10 L of water for the entire moss-growing area (72 m^2^), totalling 30 L per day and 150 L per week. Fertilization was performed once per week using modified Knop’s medium supplemented with ammonium nitrate, following Heck et al. (2021) [12]. On the fertilisation day, the room was misted three times with 10 L of nutrient solution per application.

## Protocol validation

To validate the cultivation protocol described above, four moss species were grown over four months (June–September 2025). During the validation phase, several climate-related parameters were adjusted iteratively – including watering cycle, ventilation intensity, and fertilization frequency – until stable establishment and acceptable growth were achieved. All mosses were cultivated on the same type of non-woven hemp fiber substrate to ensure comparability across treatments.

**Fig. 3:**
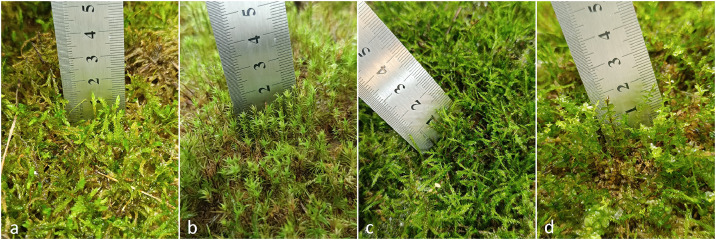
Growth of moss species on hemp mats after a four-month cultivation period: (a) *Pleurozium schreberi,* (b) *Dicranum scoparium,* (c) *Hypnum cupressiforme,* (d) *Plagiomnium affine*.

By the end of the cultivation period, most hemp tiles achieved over 80% surface coverage, with an average moss height of 1–2 cm (Fig. 3). Moss vitality and growth success were assessed visually, primarily by detecting new shoot formation and estimating the length of newly formed tissue. Biomass was not measured, and no destructive sampling was performed, as the primary aim was to evaluate practical establishment potential rather than quantify production yield.

Among the tested species, *Plagiomnium affine* exhibited the fastest initial growth, reaching approximately 2 cm within 2–3 months, but declined after the third month, particularly in monoculture. When co-cultivated, *P. affine* maintained prolonged survival, likely due to interspecific interactions that enhanced local microclimatic buffering. In particular, *Hypnum cupressiforme* frequently filled air gaps between shoots of neighbouring mosses, reducing desiccation risk and stabilizing humidity around the gametophytes. *H. cupressiforme* colonized low-density areas effectively and regenerated in mould-affected zones, making it the most resilient under the tested conditions. *Pleurozium schreberi* performed best at higher planting densities or in mixed culture, whereas *Dicranum scoparium* required very high density to maintain vigor. At lower densities, *D. scoparium* frequently turned yellow and died. These observations were qualitative in nature; no statistical analyses were performed, and no numerical growth measurements (e.g., biomass increase) were collected which represents a methodological limitation of this validation stage.

Fungal and microbial infections were the primary causes of moss decay. Mosses were sprayed with hydrogen peroxide twice per week, with a three-day interval between treatments. Throughout the trial, H_2_O_2_ concentrations of 0.25%, 0.5%, 0.75%, 1.0%, 1.25%, 1.5%, 1.75% and 2.0% were tested sequentially, increasing the concentration each week. Concentration ≥1.5% caused visible chlorosis, and higher concentrations intensified discoloration; therefore treatments above 1.25% are not recommended. Although the 1.25% solution slowed mould development, it did not fully suppress fungal spread

The validation results reveal a clear trade-off between rapid colonization and long-term robustness. This contrast can be interpreted within the CSR ecological framework [16,17], which categorizes plant strategies along the competitor (C), stress-tolerator (S), and ruderal (R) axes. The model provides a functional perspective on species behaviour by linking growth rate, persistence, and stress resilience to their dominant adaptive strategies. Within this context, *P. affine* aligns with a mixed competitor-ruderal (CR) strategy, characterized by rapid colonization, efficient resource use, and an ability to exploit favourable conditions, yet combined with comparatively low tolerance to prolonged stress or disturbance. By contrast, *H. cupressiforme* exhibits traits typical of stress-tolerant (S) species, showing slower growth but high resilience, robustness under suboptimal conditions, and strong recovery capacity following disturbance. Applying the CSR framework to these qualitative observations supports practical decision-making for cultivation planning, even in the absence of quantitative trait measurements:

1. High-risk, fast-return option: Use a fast-growing CR-type species such as *P. affine* (preferably in a mixed culture) to achieve rapid production cycle, accepting a higher risk of crop loss due to disease and environmental fluctuations.
2. Low-risk, slower-return option: Cultivate S-type resilient species such as *H. cupressiforme* to ensure a stable and predictable, albeit slower, production cycle.

Thus, the "best" species choice depends on the producer's risk tolerance, desired turnover time, and management capacity. This framework transforms the validation results from a descriptive assessment into a strategic cultivation model grounded in ecological theory.

In summary, the results demonstrate that the protocol reliably supports moss establishment and growth under controlled conditions, is reproducible across multiple cultivation units, and is suitable for scaled-up application. The method proved most robust for *H. cupressiforme* and *P. affine* (in mixed culture) while *P. schreberi* and *D. scoparium* require careful optimization of initial planting density and mould control measures (Table 1). The absence of numeric data and statistical analysis represents a limitation but does not undermine the practical conclusions regarding species performance under the tested conditions.

**Table 1:** **.** Comparative analysis of four moss species performance in a non-sterile cultivation protocol.

Moss species	Growth rate (initial)	Resistance to mould	Recommended planting density	Cultivation considerations	Overall suitability for large-scale cultivation
*Hypnum cupressiforme*	Moderate	High	Moderate to high	Grows well in monoculture; low maintenance	High
*Plagiomnium affine*	High	Low	High	Performs best in mixed culture	Medium
*Pleurozium schreberi*	Moderate	Medium	High	Requires high density or mixed culture	Medium
*Dicranum scoparium*	Slow	Very low	Very high	Sensitive to contamination; needs dense planting	Low

## Risk assessment: Pollution and mitigation strategies

In a non-sterile system using wild-harvested starting material, contamination is a predictable and manageable factor. The reactive chemical treatment used in the protocol is only a short-term solution, not a sustainable strategy. It slows the spread of mould but does not eliminate the cause and can also stress the mosses if the concentration is too high. The authors will continue to research possible long-term solutions. This assessment ensures compliance with sustainable cultivation principles and supports safe implementation in both research and commercial facilities.

## Limitations

Cultivating mosses under controlled climatic conditions presents several challenges due to complex interplay of environmental and biological factors. Key variables requiring careful optimization include mineral nutrition, relative humidity, temperature, light quality and intensity, photoperiod, substrate composition, and potential symbiotic interactions with microorganisms. A consistently high humidity level is essential for successful moss cultivation; however, elevated humidity also promotes mould and fungal growth, which can negatively impact both the moss cultures and the cultivation facility. In addition to mould, naturally occurring fungi from forest-collected moss samples may proliferate under humid conditions, further complicating cultivation and potentially affecting moss vitality. Since mould growth on indoor surfaces can cause structural damage, greenhouse environments are recommended over enclosed indoor spaces for moss cultivation. Future work should explore automated humidity management and alternative substrates to improve scalability and reduce contamination risks.

This method also necessitates a transition to specialised agricultural infrastructure. Purpose-built greenhouses are required to accommodate high humidity, offering improved ventilation, drainage, and moisture and mould resistant materials. This shifts the approach from a potentially low-cost indoor solution to a higher-cost agricultural enterprise, demanding greater capital investment.

### Guidelines for practitioners

1. Gradual introduction: Begin with the most resilient species, *Hypnum cupressiforme*, to learn system dynamics and management practices before introducing more sensitive species.
2. Moss processing: Thoroughly clean moss samples to remove organic debris (e.g., needles, twigs). Carefully calibrate the initial planting density, as this parameter is critical for successful establishment, particularly for sensitive species.
3. Proactive contamination management: Transition from reactive hydrogen peroxide treatments to an integrated pest management (IPM) approach. This may include the introduction of beneficial microorganisms (e.g., probiotics) that outcompete mould, or the use of mould-feeding microfauna (e.g., *Collembola*). Optimizing airflow and filtration using UV air recirculators, can further reduce airborne fungal spores. Additionally, adjusting light spectra and photoperiods to create conditions less favourable for fungal growth may enhance long-term stability.

### Directions for future research

1. Closed-loop feedstock production: The current protocol relies on wild-harvested mosses, which is unsustainable for large-scale or long-term applications. Moreover, moss collection and subsequent processing (including cleaning, sorting, and preparation for cultivation) require considerable labour and time, representing a major bottleneck in production scalability. To address these limitations, a two-tier system should be developed: (1) a highly controlled, sterile or semi-sterile "mother crop" facility for producing pure feedstock, and (2) non-sterile production areas (as described in this protocol) for propagating this feedstock. Such a system would reduce dependence on wild resources and enable sustainable, standardized biomass production.
2. Automation and process optimization: The current protocol remains labour-intensive, involving frequent manual interventions such as irrigation and spraying up to three times daily. Future research should explore automation of irrigation, fertilization, and climate control through integrated sensor and microcontroller systems. Automation would not only improve reproducibility and environmental stability but also substantially reduce operational costs and human resource demands.
3. Substrate and nutrient refinement: Further investigation is needed into alternative substrates that offer greater resistance to fungal degradation or that support beneficial microbial communities. Additionally, different nutrient solutions, including modified versions of Knop’s medium, should be tested and optimized for the specific requirements of each moss species to enhance growth and persistence.

## CRediT author statement

**Liga Strazdina:** Conceptualization, Methodology, Validation, Writing – review & editing. **Lilita Abele:** Validation, Supervision, Writing – review & editing. **Roberts Krumins:** Methodology, Validation, Investigation, Data curation, Writing – original draft, review & editing, Visualization. **Juta Karklina:** Writing – review & editing, Visualization. **Gederts Ievinsh:** Writing – review & editing. **Inga Straupe:** Supervision. **Edgars Karklins:** Writing – review & editing.

## Declaration of competing interest

The authors declare that they have no known competing financial interests or personal relationships that could have appeared to influence the work reported in this paper.

## Data Availability

No data was used for the research described in the article.
